# Genotype diversity of brucellosis agents isolated from humans and animals in Greece based on whole-genome sequencing

**DOI:** 10.1186/s12879-023-08518-z

**Published:** 2023-08-14

**Authors:** Hanka Brangsch, Vassilios Sandalakis, Maria Babetsa, Evridiki Boukouvala, Artemisia Ntoula, Eirini Makridaki, Athanasia Christidou, Anna Psaroulaki, Kadir Akar, Sevil Erdenlig Gürbilek, Tariq Jamil, Falk Melzer, Heinrich Neubauer, Gamal Wareth

**Affiliations:** 1 Institute of Bacterial Infections and Zoonoses, Fredrich-Loeffler-Institut – Federal Research Institute for Animal Health (FLI), 07743 Jena, Germany; 2 Laboratory of Clinical Microbiology and Microbial Pathogenesis, School of Medicine, University of Crete, Heraklion, Crete, 71500 Greece; 3 Veterinary Research Institute, ELGO-DIMITRA, Campus of Thermi, Thermi, 57001 Thessaloniki Greece; 4 Faculty of Veterinary Medicine, Van Yuzuncu Yıl University, Van, 65090 Turkey; 5 Microbiology Department, Faculty of Veterinary Medicine, Harran University, Şanlıurfa, 63200 Turkey; 6 Institute of Infectious Diseases and Infection Control, Jena University Hospital, 07747 Jena, Germany

**Keywords:** Brucellosis, Greece, *Brucella melitensis*, East Mediterranean lineage, *Brucella abortus* RB51, Virulence genes, cgSNP, cgMLST

## Abstract

**Background:**

Brucellosis is a zoonotic disease whose causative agent, *Brucella* spp., is endemic in many countries of the Mediterranean basin, including Greece. Although the occurrence of brucellosis must be reported to the authorities, it is believed that the disease is under-reported in Greece, and knowledge about the genomic diversity of brucellae is lacking.

**Methods:**

Thus, 44 *Brucella* isolates, primarily *B. melitensis*, collected between 1999 and 2009 from humans and small ruminants in Greece were subjected to whole genome sequencing using short-read technology. The raw reads and assembled genomes were used for *in silico* genotyping based on single nucleotide substitutions and alleles. Further, specific genomic regions encoding putative virulence genes were screened for characteristic nucleotide changes, which arose in different genotype lineages.

**Results:**

*In silico* genotyping revealed that the isolates belonged to three of the known sublineages of the East Mediterranean genotype. In addition, a novel subgenotype was identified that was basal to the other East Mediterranean sublineages, comprising two Greek strains. The majority of the isolates can be assumed to be of endemic origin, as they were clustered with strains from the Western Balkans or Turkey, whereas one strain of human origin could be associated with travel to another endemic region, e.g. Portugal. Further, nucleotide substitutions in the housekeeping gene *rpoB* and virulence-associated genes were detected, which were characteristic of the different subgenotypes. One of the isolates originating from an aborted bovine foetus was identified as *B. abortus* vaccine strain RB51.

**Conclusion:**

The results demonstrate the existence of several distinct persistent *Brucella* sp. foci in Greece. To detect these and for tracing infection chains, extensive sampling initiatives are required.

**Supplementary Information:**

The online version contains supplementary material available at 10.1186/s12879-023-08518-z.

## Background

Members of the genus *Brucella* are facultative intracellular pathogens causing brucellosis, a neglected zoonotic disease which was initially described in soldiers stationed in Malta. The *Brucella* (*B*.) spp. host spectrum primarily comprises domestic animals, like small ruminants, cattle or dogs, with species-specific preferences, while humans may become accidental hosts [1–3]. The prevalence of brucellosis tremendously varies between different areas worldwide, but countries of the Mediterranean basin are among those with the highest incidences [4], especially compared to Northern European countries. In the European Union, most countries are free of caprine and ovine brucellosis, and most human cases are due to traveling to endemic regions, with the exception of some countries in the South, e.g. Portugal, Italy and Greece, where brucellosis is still endemic [1, 4, 5]. Among these countries, Greece reported the highest notification rate of human brucellosis cases in 2019 (0.61 cases per 100,000 population), exceeding the European average by a factor of 10. However, a downward trend in brucellosis incidence has been observed since 2014 [4]. Accordingly, none of the Greek regions is officially free of bovine, ovine, and caprine brucellosis, although prevalence varies among Greek provinces [6]. To fight the disease, Greece was divided into two zones in 2004 based on the prevailing brucellosis prevention measures: the vaccination zone and the eradication zone [6, 7]. The latter includes most of the Greek islands, where a surveillance system has been established with regular testing and slaughter of brucellosis positive animals. The mainland and some islands have been declared a vaccination zone where female small ruminants are vaccinated with *B. melitensis* strain Rev-1, while male animals are tested annually [6, 7]. Large year-to-year variations in prevalence in small ruminants have been reported, with 8.6% in 2012 and 0.4% in 2015. In 2019, the prevalence decreased to 0.16% [4]. Despite the fact that notification of brucellosis cases is mandatory in most EU Member States, several studies documented under-reporting of the disease in Greece, e.g., due to nonspecific disease symptoms complicating diagnosis [6–10].

Brucellosis is predominantly an occupational disease that primarily affects professionals handling animals, e.g. veterinarians, livestock keepers, and breeders, who contract the disease via direct contact with infected animals. However, the ingestion of raw, unpasteurized dairy products also poses a risk of *Brucella* infection [4, 6, 8, 11, 12]. In Europe, particularly in northern countries, brucellosis is primarily associated with travellers or immigrants from endemic regions and importation of goods and animals, e.g., from the Middle East, which indicates that globalization and the increase in travelling has altered brucellosis transmission routes, posing the risk of introduction into regions considered free of the disease [5, 13].

Thus, determining the origin of an outbreak strain is of paramount importance. However, due to the highly conserved nature of *Brucella* genomes and a low genetic variability between strains, sensitive detection and genotyping methods are needed [13, 14]. Multilocus variable number of tandem repeats analyses (MLVA) and multilocus sequence typing (MLST) both target a relatively small number of target genes and allow differentiation of strains to a certain degree [15–17]. Higher resolution can be achieved by using whole-genome sequencing (WGS)-based techniques, such as the analysis of single nucleotide polymorphisms (SNPs) or thousands of target genes in a core genome MLST (cgMLST) approach [18]. Based on genomic analysis, five major *B. melitensis* genotypes, the main causative agent of brucellosis in Greece [6, 7, 19], have been identified, which in turn include several subgenotypes [20]. In addition, these methods allow linkage of human brucellosis cases to *Brucella* strains isolated from animals or their products [11]. Nucleotide variations in the housekeeping gene *rpoB*, which encodes the β subunit of the DNA-dependent RNA polymerase, were also found to be reliable markers for distinguishing the different *B. melitensis* lineages and biovars [13, 21]. Additionally, mutations in *rpoB* could confer resistance to rifampin, an antibiotic used to treat brucellosis [22, 23]. It should be noted that with the advent of sequencing methods, the differentiation of *Brucella* strains in biovars has become virtually obsolete, as genotyping has shown that biovars do not correlate with actual phylogenetic relationships of *Brucella* strains [15, 24].

Although brucellosis is endemic in Greece, little is known about the local genomic diversity of *Brucella* strains. Therefore, in this study, isolates from humans and animals from a period of 11 years were analysed for assessing phylogenetic and epidemiological connections. Further, by determining the presence and potential mutations in antimicrobial resistance and virulence genes, a first step towards monitoring of prevailing evolutionary changes in *Brucella* spp. shall be taken, as the acquisition of increased virulence or resistance could promote the spread of a particular *Brucella* lineage.

## Methods

### Strain isolation and biotyping

In the current study, a total of 44 *Brucella* isolates were examined (Table 1), of which 28 were recovered from small ruminants (sheep and goats), one isolate from an aborted bovine foetus, and 15 human isolates. The latter were obtained from blood samples, except for one that was recovered from cerebrospinal fluid (CSF). All samples were aseptically inoculated into liquid blood cultures. The inoculated media were incubated at 37 °C for a maximum of 30 days. Broth cultures were periodically sampled for culture on solid media. At the time of isolation, all colonies were analysed based on morphology, CO_2_ requirement, H_2_S production, as well as oxidase, catalase, and urease activity. Further, basic fuchsin and thionin sensitivity tests, lysis by *Brucella* phages (Tbilisi (Tb), Berkeley (Bk), Izatnagar (Iz) and Weybridge (Wb)) were conducted and agglutination in monospecific anti-sera A and M was tested, all as described by Christoforidou et al. [25].

**Table 1:** *Brucella* strains isolated from animals and human between 1999 and 2010 in Greece

Strain	Species	Host	Source	Date	Biovar
Bm-GRC-A1h	*B. melitensis*	Human	Blood	2006	3
Bm-GRC-A2h	*B. melitensis*	Human	Blood	2005	3
Bm-GRC-A3h	*B. melitensis*	Human	Blood	2006	3
Bm-GRC-A4h	*B. melitensis*	Human	Blood	2000	3
Bm-GRC-A5h	*B. melitensis*	Human	Blood	2000	3
Bm-GRC-A6h	*B. melitensis*	Human	Blood	2000	2
Bm-GRC-A7h	*B. melitensis*	Human	Blood	1999	3
Bm-GRC-A8h	*B. melitensis*	Human	Blood	2000	3
Bm-GRC-A9h	*B. melitensis*	Human	Blood	1999	2
Bm-GRC-B2h	*B. melitensis*	Human	Blood	1999	3
Bm-GRC-B9h	*B. melitensis*	Human	Blood	2000	3
Bm-GRC-C1h	*B. melitensis*	Human	Blood	2006	3
Bm-GRC-C5h	*B. melitensis*	Human	Blood	2009	3
Bm-GRC-C6h	*B. melitensis*	Human	Blood	2000	3
Bm-GRC-D9h	*B. melitensis*	Human	CSF	1999	3
Bm-GRC-T2s	*B. melitensis*	Sheep	Liver	1999–2010	2
Bm-GRC-T3g	*B. melitensis*	Goat	Liver	1999–2010	1
Bm-GRC-T4s	*B. melitensis*	Sheep	no data	1999–2010	1
Bm-GRC-T5s	*B. melitensis*	Sheep	Spleen	1999–2010	3
Bm-GRC-T6s	*B. melitensis*	Sheep	no data	1999–2010	3
Bm-GRC-T8g	*B. melitensis*	Goat	Liver	1999–2010	3
Bm-GRC-T9s	*B. melitensis*	Sheep	Liver	1999–2010	3
Bm-GRC-T10s	*B. melitensis*	Sheep	no data	1999–2010	3
Bm-GRC-T13s	*B. melitensis*	Sheep	Liver	1999–2010	3
Bm-GRC-T14s	*B. melitensis*	Sheep	Foetus	1999–2010	3
Bm-GRC-B2s	*B. melitensis*	Sheep	no data	1999–2010	1
Bm-GRC-B7s	*B. melitensis*	Sheep	no data	1999–2010	1
Bm-GRC-B16s	*B. melitensis*	Sheep	no data	1999–2010	1
Bm-GRC-B20s	*B. melitensis*	Sheep	no data	1999–2010	3
Bm-GRC-B21s	*B. melitensis*	Sheep	no data	1999–2010	1
Bm-GRC-B22s	*B. melitensis*	Sheep	no data	1999–2010	3
Bm-GRC-B24s	*B. melitensis*	Sheep	no data	1999–2010	3
Bm-GRC-B26s	*B. melitensis*	Sheep	no data	1999–2010	1
Bm-GRC-B28s	*B. melitensis*	Sheep	no data	1999–2010	3
Bm-GRC-B30s	*B. melitensis*	Sheep	no data	1999–2010	3
Bm-GRC-B31s	*B. melitensis*	Sheep	no data	1999–2010	3
Bm-GRC-B32s	*B. melitensis*	Sheep	no data	1999–2010	3
Bm-GRC-B33s	*B. melitensis*	Sheep	no data	1999–2010	3
Bm-GRC-B34s	*B. melitensis*	Sheep	no data	1999–2010	3
Bm-GRC-B35s	*B. melitensis*	Sheep	no data	1999–2010	3
Bm-GRC-B36s	*B. melitensis*	Sheep	no data	1999–2010	3
Bm-GRC-B37s	*B. melitensis*	Sheep	no data	1999–2010	3
Bm-GRC-B38s	*B. melitensis*	Sheep	no data	1999–2010	3
Ba-GRC-O1c	*B. abortus*	Cattle	Foetus	2018	-

### DNA extraction and genome sequencing

For whole-genome sequencing, DNA was extracted using the High Pure PCR Template Preparation Kit (Roche Molecular Systems, Pleasanton, CA, USA) and sequencing libraries were prepared using the Nextera XT library preparation kit (Illumina Inc., San Diego, CA, USA), all according to the manufacturers’ recommendations. Paired-end sequencing was conducted on a MiSeq system using v3 chemistry (Illumina Inc., San Diego, CA, USA) for 2 × 300 bp long reads.

### Sequencing quality control and genome assembly

The raw sequencing reads were processed as follows: read quality was assessed by FASTQC v0.11.7 (https://www.bioinformatics.babraham.ac.uk/projects/fastqc/) and kraken2 v2.0.7_beta [26] was used for checking for contaminations and confirming the species identity. From the reads, genomes were assembled in a de novo approach using Shovill v.1.0.4 (https://github.com/tseemann/shovill), including adapter sequence trimming from the reads by trimmomatic, stitching of paired-end reads and subsequent assembly with Spades as implemented in Shovill. The quality of the resulting assemblies was subsequently assessed by QUAST v5.0.2 [27] and coding regions predicted by Prokka v.1.14.5 [28].

### Genotyping based on WGS data

Based on the assemblies, MLST and MLVA-16 were conducted *in silico* using mlst v2.19.0 (https://github.com/tseemann/mlst) employing the PubMLST website [29] and MISTReSS (https://github.com/Papos92/MISTReSS) with the scheme by Le Flèche et al. [16] and Al Dahouk et al. [15]. The MLVA profiles were compared to published profiles of Greek *Brucella* spp. deposited in MLVAbank (https://microbesgenotyping.i2bc.paris-saclay.fr/ [30], accessed on 21.09.2022) and a minimum spanning tree was calculated using GrapeTree [31]. Further, the new *B. melitensis* assemblies were analysed by cgMLST with Ridom SeqSphere + v7.7 [32] applying the scheme by Janowicz et al. [18]. A minimum spanning as well as neighbour joining tree was generated based on the allelic differences as implemented in Seqsphere + with pairwise ignoring missing values. For cluster detection, the minimum allele identity was set to 6, as defined by Janowicz et al. [18].

Additionally, SNP analysis was chosen as an assembly-independent genotyping approach. SNPs were called by Snippy v.4.6.0 (https://github.com/tseemann/snippy) in default mode. Beforehand, a *B. melitensis* reference strain was chosen by comparing the Mash distances of the new genome assemblies with published *B. melitensis* genome assemblies using Mash v2.1.1 [33]. Based on this analysis, *B. melitensis* M28 (GCF_000192725.1) and *B.* abortus 2308 (GCF_000054005.1) were chosen as references for SNP detection. However, for assigning the Greek *B. melitensis* strains to the established global genotypes, *B. melitensis* 16 M (GCF_000007125.1) was used as a reference. From the detected SNPs, a core genome alignment was created by Snippy and the number of different SNPs was counted using snp-dists v0.7.0 (https://github.com/tseemann/snp-dists). Further, the alignment served as input for maximum likelihood analysis using RAxML v8.2.12 [34] with the GTRGAMMA model of rate heterogeneity and optimization of substitution rates. The tree was visualized by FigTree v1.4.3 (http://tree.bio.ed.ac.uk/software/figtree/).

In the SNP analysis, also raw read data of *B. melitensis* and *B. abortus* deposited in the NCBI Sequence Read Archive (SRA) (accessed on 20.08.2022) were included. The quality of this data was first controlled as described above.

Dendroscope v3.5.9 [35] was used for comparing the clustering generated by cgMLST and cgSNP analysis.

### AMR and virulence gene detection

For targeting the problem of antimicrobial resistances, the Greek assembled genomes were screened using Abricate v0.8.10 (https://github.com/tseemann/abricate) with entries from the databases Resfinder [36], CARD [37] and NCBI’s AMRFinder [38] Additionally, mutations in suspected virulence-associated genes and the housekeeping gene *rpoB*, compared to the reference strain *B. melitensis* 16 M (GCF_000007125.1), were analysed. Deviations in the *rpoB* gene were detected using snpEff v5.0e [39], as implemented in Snippy, which also predicts effects on the coded gene product, i.e. amino acid substitutions. *In silico* PCR for 15 potential virulence genes was conducted using a script by Egon A. Ozer (v0.5.1) (https://github.com/egonozer/in_silico_pcr) with primers by Hashemifar et al. [40] and Mirnejad et al. [41] (Table 2). The respective products were aligned using MAFFT v7 [42], base substitutions were determined and potential effects on the gene products were assessed by snpEff as before.

**Table 2:** Primers used for *in silico* amplification of virulence-associated genes [40, 41]. Locus designation and product size according to reference strain *B. melitensis* 16 M (GCF_000007125.1).

Target gene	Locus tag	Primers	Product size
*omp19*	BME_RS00635	5‘-TGA TGG GAA TTT CAA AAG CA-3‘ 5‘-GTT TCC GGG TCA GAT CAG C-3‘	550
*wbkA*	BME_RS07060	5‘-AAT GAC TTC CGC TGC CAT AG-3‘ 5‘-ATG AGC GAG GAC ATG AGC TT-3‘	931
*manA*	BME_RS07015	5‘-TCG ATC CAG AAA CCC AGT TC-3‘ 5‘-CAT ACA CCA CGA TCC ACT GC-3‘	271
*mviN*	BME_RS08945	5‘-GCA GAT CAA CCT GCT CAT CA-3‘ 5‘-GGC CAT AGA TCG CCA GAA TA-3‘	344
*ure*	BME_RS03220 BME_RS03225 BME_RS03230	5‘-GCT TGC CCT TGA ATT CCT TTG TGG-3‘ 5‘-ATC TGC GAA TTT GCC GGA CTC TAT-3‘	2214
*perA*	BME_RS07090	5‘-GGA ACG GTG GCA CTA CAT CT-3‘ 5‘-GGC TCT CTG TGT TCC GAG TT-3‘	716
*virB5*	BME_RS10335	5‘-ATT CTC AGC TTC GCA TTC-3‘ 5‘-TCA CCG CTT CGT AGA GAT-3‘	274
*btpA*	BME_RS08335	5‘-CTA TCA GGC TAA GCA ATT C-3‘ 5‘-CGT AGG AAA CTT TAT GCC-3‘	458
*btpB*	BME_RS06120	5‘-TTA ACC AGC ACG AAT ACA CG-3‘ 5‘-CTA CGA TCA GTT TGC AGC G-3‘	579
*vceC*	BME_RS04715	5‘-CGC AAG CTG GTT CTG ATC-3‘ 5‘-TGT GAC GGG TAA TTT GAA GC-3‘	482
*betB*	BME_RS06955	5‘-GCT CGA AAC GCT GGA TAC-3‘ 5‘-AGG CGA TGA TTG ACG AGC-3‘	393
*bpe275*	BME_RS03675	5‘-TGT CGC GGT CTA TGT CTA TC-3‘ 5‘-AAT GAG GAC GGG CTT GAG-3‘	466
*virB2*	BME_RS10320	5‘-GCT GTC GCG GAT TCT ACC-3‘ 5‘-CGG AAT GCC ATC TTG TAA C-3‘	198
*bspB*	BME_RS06340	5‘-TAT CCA TGG TAT ATG CGC C-3‘ 5‘-ATA AAG GCC GGG AAT GAC-3‘	336
*prpA*	BME_RS01255	5‘-AAC CTC AAT GGA TCG ACC-3‘ 5‘-ACG GTC GAT AGC CTT GTC-3‘	672

### Strain identity verification for ***B. abortus***

In order to verify the identity of the Greek *B. abortus* isolate, the assembled contigs of the isolate were mapped to the genome assembly of vaccine strain *B. abortus* RB51 (GCF_011801185.1) using minimap2 v2.22-r1101 [43]. Also, the raw reads mapped to the same reference by Snippy, which employed BWA v0.7.17 [44], were examined. Both mappings were visualized in IGV v2.3.92 [45]. Further, a partial Bruce-ladder PCR using primer pairs BMEI0998f (5‘-CCC CGG AAG ATA TGC TTC GAT CC-3‘) and BMEI0997r (5‘-TGC CGA TCA CTT AAG GGC CTT CAT-3‘) [46] was conducted with the *in silico* PCR script mentioned above to differentiate *B. abortus* RB51 from its ancestor *B. abortus* 2308.

### Data availability

Raw sequencing reads were submitted to the European Nucleotide Archive (ENA) and can be accessed under the project number PRJEB56772.

## Results

### Biotyping-based strain identification and genome sequencing

Out of 44 investigated *Brucella* strains, all 15 isolates originating from humans from southern Greece were identified as *B. melitensis*. Likewise, all strains isolated from sheep (n = 26) and two goats in the northern parts of Greece belonged to this species. Thirty-three *B. melitensis* strains (76.7%) had been identified as biovar 3, while seven strains (16.3%) belonged to biovar 1, and only three strains (6.4%) belonged to biovar 2 (Table 1). The isolate Ba-GRC-O1c from an aborted bovine foetus was the only investigated isolate identified as *B. abortus* (Table 1), i.e. vaccine strain RB51.

Draft genomes obtained by *de novo* assembly comprised 23–54 contigs adding up to genomes of 3,289,118 bp to 3,293,386 bp for the *B. melitensis* isolates and 3,262,813 bp in the case of *B. abortus* Ba-GRC-O1c, accounting for > 99% of the respective reference genome (Additional file 1). Between 3,110 and 3,143 coding sequences could be detected and the GC content of all isolates varied between 57.24 to 57.26%GC.

### Allele-based genotyping

In the MLST analysis, all Greek *B. melitensis* strains were assigned to sequence type (ST) 8, and *B. abortus* Ba-GRC-O1c was identified as ST5. A higher resolution was achieved by using MLVA. Here, 23 different allelic profiles were found for the Greek *B. melitensis* (see Additional file 2), most of which were novel profiles when compared to the entries in the MLVAbank. No identical profiles between human and animal isolates were found, although the allelic differences between strains were mostly comparably low (0–7 alleles). The largest cluster of identical strains comprised eight isolates, seven isolated from sheep and one from a goat. When comparing the investigated strains to other Greek strains (Additional file 3), three foreign isolates were found to be identical in their allelic profile to the here investigated strains: an isolate from 1990 from cattle was identical to the human isolate Bm-GRC-C5h, a 2002 human isolate (BfR_12) was identical to Bm-GRC-T14s, which was isolated from sheep and, lastly, a strain isolated from Caprinae in 1983 was identical to the cluster of eight strains mentioned before. *B. abortus* Ba-GRC-O1c markedly differed from the foreign Greek *B. abortus* isolates. The allelic profile was found to be identical to that of *B. abortus* 2308.

**Fig. 1:**
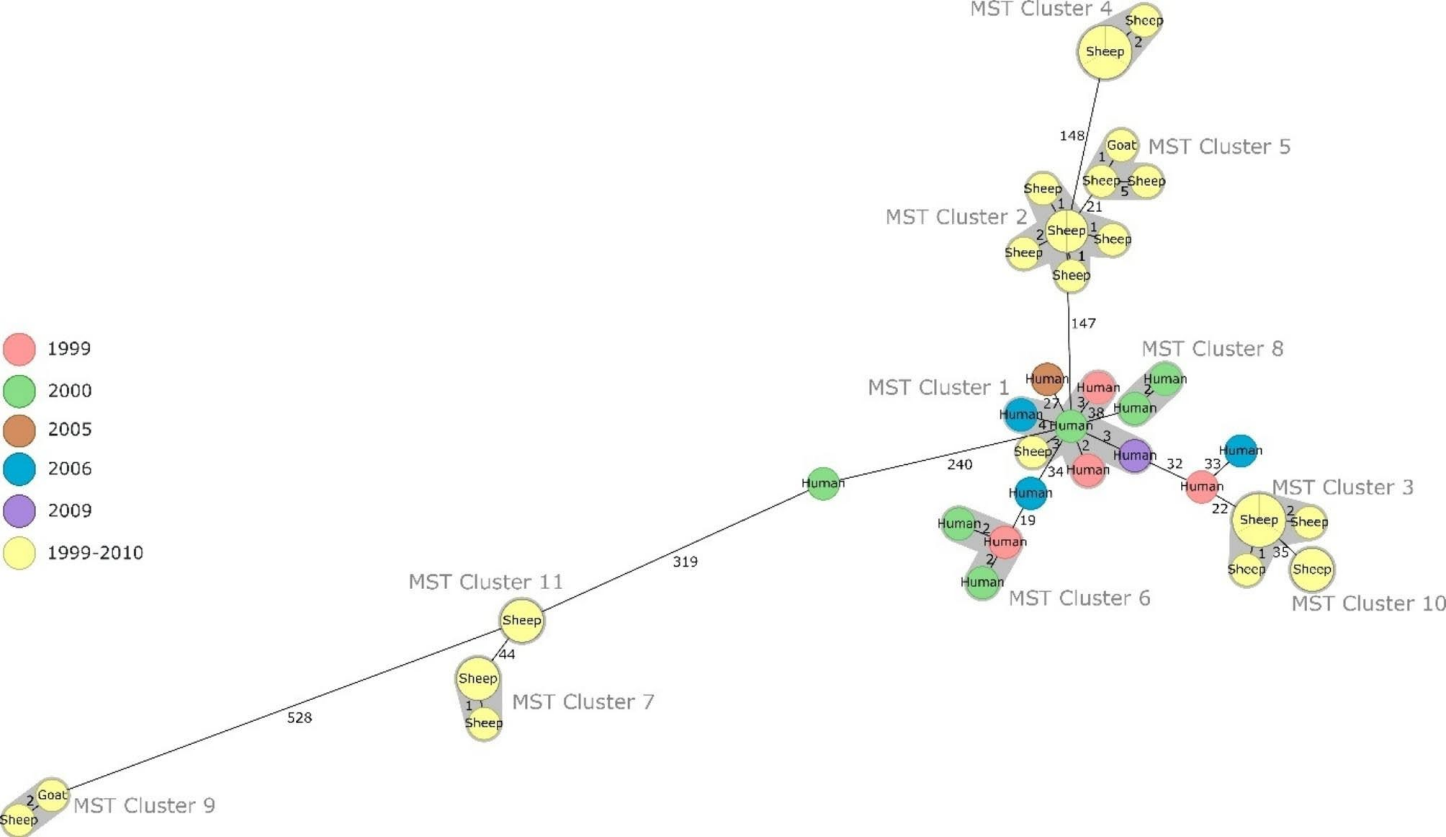
Minimum spanning tree based on allelic cgMLST differences between Greek *B. melitensis* isolates. Clusters are defined by max. 6 alleles deviation. Colours indicate the year or period of isolation

As the informative value of MLVA can be compromised by homoplasy, cgMLST was conducted for the *B. melitensis* isolates that included 2704 target genes. In that way, a higher diversity was revealed and eleven clusters of strains could be identified (Fig. 1), indicating an epidemiological link between the clustering strains. Notably, these clusters always exclusively comprised strains of either animal or human origin, with only one exception: strain Bm-GRC-B31s isolated from sheep belonged to MST cluster 1, which otherwise comprised strains isolated from humans in 1999, 2000, 2006, and 2009, displaying 3–5 alleles difference to these human isolates. MST cluster 9, which is formed by two isolates from goat and sheep markedly differed from most of the other Greek isolates, displaying 528 to 559 alleles difference.

**Fig. 2:**
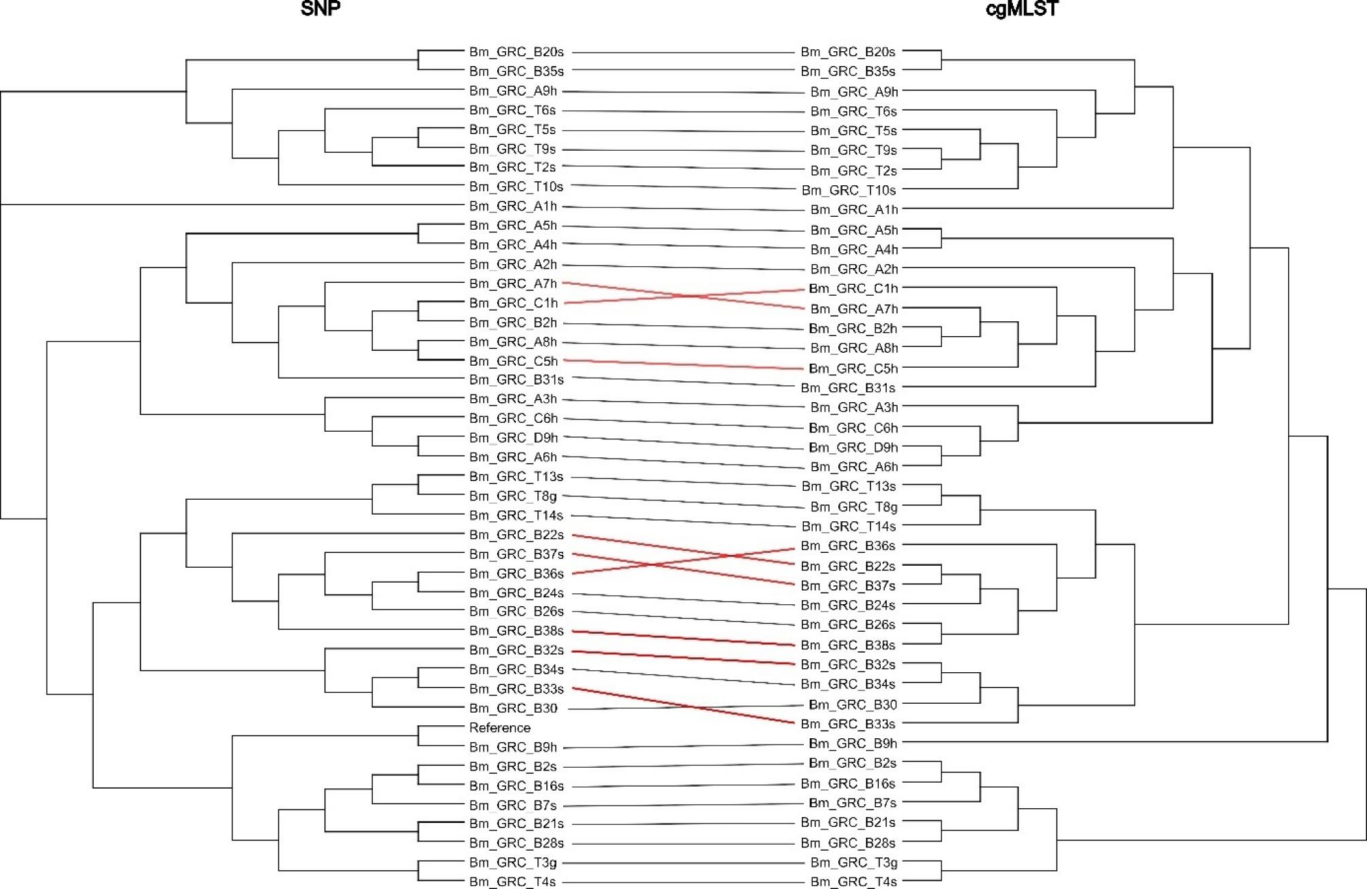
Tanglegram showing the congruence between neighbour joining tree based on cgMLST allelic differences and maximum likelihood tree based on cgSNP analysis of the Greek *B. melitensis* isolates. Lines coloured red indicate substantial differences in the placements of the strains within the clusters

In order to decide which method to use for a comprehensive comparison of the Greek isolates to foreign strains, the clustering generated by cgMLST and by cgSNP analysis was compared. The resulting trees were roughly congruent (Fig. 2). However, some of the strains grouped differently within the two largest clusters. As it can be expected that SNP typing provides a higher discriminatory level, it was decided to follow the cgSNP typing approach for placing the strains within the global phylogeny and similarity analysis to foreign strains.

### SNP-based genotyping for ***B. melitensis*** isolates

**Fig. 3:**
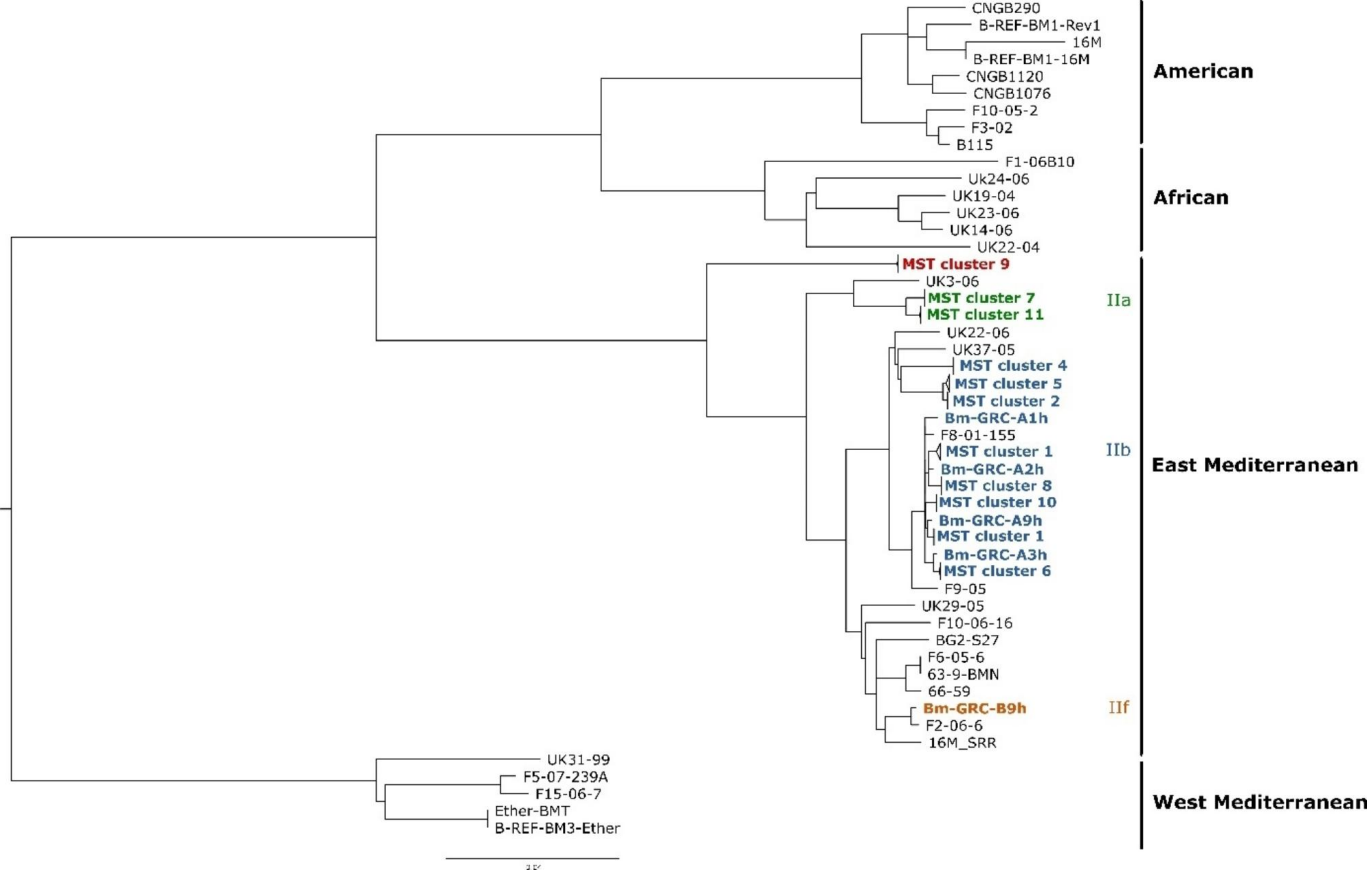
Maximum likelihood tree generated by cgSNP alignment of strains from the main genotype groups of *B. melitensis*. Branches containing strains assigned to the same MST cluster by cgMLST are collapsed and named according to cgMLST. Colours indicate the different genotypes detected for the Greek *B. melitensis* isolates. The scale bar gives the number of base substitutions per site

To determine the affiliation of the Greek *B. melitensis* strains to the known genomic groups of this species, SNPs compared to the reference genome of *B. melitensis* 16 M were determined and compared with members of the major phylogenetic groups (Fig. 3). The MST clusters defined by cgMLST analysis could be also found in the SNP analysis, where these strains formed clusters. The Greek isolates belonged to three different genotypes (IIa, IIb, IIf) within the East Mediterranean lineage. Remarkably, two strains forming MST cluster 9 that also showed the highest deviation from the other isolates in cgMLST analysis, formed a separate branch in the tree, which was basal to the other branches of the East Mediterranean strains and could not be assigned to a sub-genotype. However, the majority of strains belonged to the sub-group IIb, subdivided into two different branches.

**Fig. 4:**
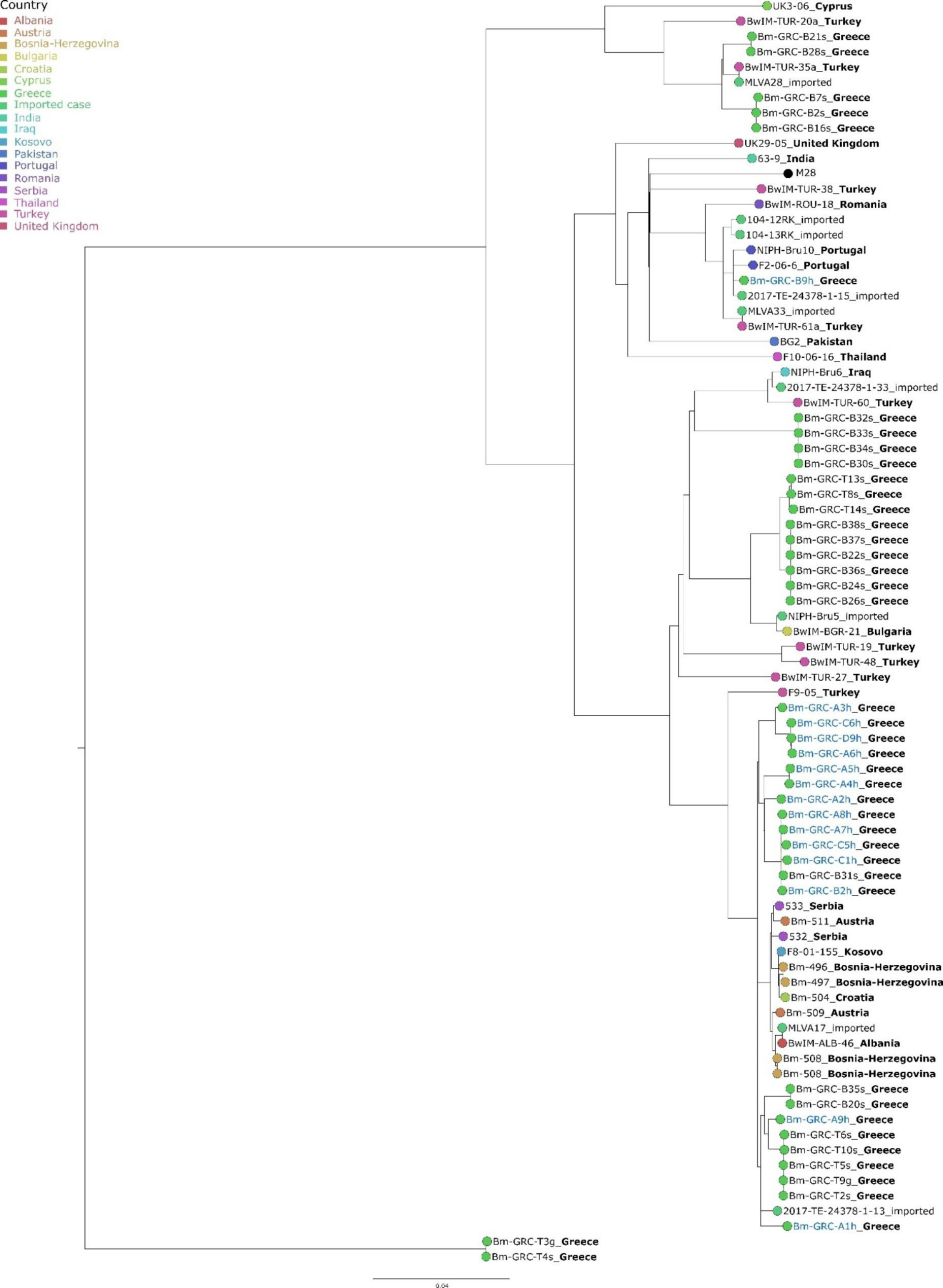
Maximum likelihood tree based on cgSNP alignment of the Greek *B. melitensis* isolates and foreign strains of the East Mediterranean genotype. Greek isolates originating from humans are coloured blue. For each strain, the country of origin is given, if known, by coloured branch tips and appended to the strain name. The scale bar indicates the number of base substitutions per site

The result of this analysis was utilized for a more in-depth comparison of the strains. Based on the classification as East Mediterranean group strains, it was decided to choose a more closely related reference strain for SNP typing than the commonly used reference strain 16 M, which belonged to the American lineage. Mash distances of the reference strains *B. melitensis* 16 M, *B. melitensis* M28 and a *B. melitensis* isolate from Albania (BwIM-ALB-46) to the genomes of seven representative Greek isolates were compared. Based on the low mash distance (< 0.001) and higher assembly contiguity, *B. melitensis* M28 was chosen as the reference genome. Further, 39 foreign strains from public repositories were included in the analysis, mostly isolated from Eastern Europe, Turkey, and imported brucellosis cases in Austria and Sweden (see Additional file 4). All in all, 3474 SNPs were called. Based on the finding of the SNP analysis beforehand, the resulting tree was rooted with the branch comprising the Greek isolates of MST cluster 9 (Fig. 4), as these were basal to the East Mediterranean group. Between these two strains, which originated from goat and sheep, there was one SNP difference and none of the chosen foreign strains showed a high similarity to these isolates. The majority of the human isolates clustered together in a branch of the IIb genotype with strains mostly isolated from Western Balkans, e.g. Bosnia-Herzegovina, Croatia, and Serbia, but also imported brucellosis cases of uncertain origin from Austria. Again, the sheep isolate Bm-GRC-31s fell in one of the clusters of human strains, exhibiting merely 3–4 SNPs difference to isolates from 1999 to 2009. The largest clusters of Greek isolates of animal origin were located on a different IIb genotype branch, with strains from Bulgaria and Turkey being the closest matches. Bm-GRC-A9h, which is also of human origin, clustered with isolates from animals as well, however, the number of SNP differences to these isolates amounted to 27 up to 42. Most of these isolates were found in sheep, except for one strain which originated from a goat and displayed a 0–1 SNP difference from the sheep’s isolates.

A single Greek human isolate, Bm-GRC-B9h, from 2006 differed markedly from the other Greek strains, as it was the sole strain belonging to genotype IIf and it was associated with isolates from Portugal from 2006 to 2009 to which it exhibited 29 and 30 SNPs differences, respectively, and even fewer SNPs (n = 20) to an imported case from Sweden in 2002.

Lastly, two Greek clusters, accounting for five strains isolated from sheep, were located at the same branch (genotype IIa) as an isolate from Cyprus and two isolates from Turkey.

### AMR and virulence gene analysis

No dedicated antimicrobial resistance genes were detected in all investigated strains when screening the assemblies for entries in three different databases (NCBI, CARD, Resfinder). However, all Greek *B. melitensis* strains exhibited mutations in the *rpoB* gene compared to the reference strain *B. melitensis* 16 M (Table 3). All strains had a single substitution (G3927A) in common. In the two strains of MST cluster 9, this was the only substitution detected in all loci examined. Strains of the IIf genotype further exhibited changes in base positions 2954 and 1886, both resulting in amino acid changes from alanine to valine. The latter substitution was shared by strains of genotype IIb. In the Greek isolates belonging to genotype IIa there was a silent mutation in base 1332 of the *rpoB* gene.

**Table 3:** Base substitutions in the *rpoB* gene of Greek *B. melitensis* isolates, relative to the reference strain *B. melitensis* 16 M, including the predicted effect on the gene product. Base and amino acid numbers refer to the reference genome

Change	Effect	Number of strains	Genotype group
A1332G	silent	5	IIa
C1886T	Ala629Val	36	IIb, IIf
C2954T	Ala985Val	1	IIf
G3927A	silent	43	IIa, IIb, IIf, novel

Additionally, 15 presumed virulence-associated genes were screened for mutations compared to the reference strain *B. melitensis* 16 M. In every investigated isolate the selected potential virulence genes were detected. While the majority of these genes was conserved compared to the reference, base substitutions were detected in seven genomic loci, comprising five genes or gene clusters: *ure*, *bspB*, *prpA*, *vceC*, and *virB2*. All isolates shared a substitution in *virB2* (BME_RS10320) (G54A, silent) and changes in *ure* at position 877 (C > T; Val293Met) in the alpha subunit and at position 222 (A > G, silent) in the beta subunit (BME_RS03225) as well as an insertion of TT between positions 12 and 13 that caused a frameshift. Further, several unique mutations were detected (Table 4). These were characteristic of some clusters detected by cgMLST and SNP typing, but not necessarily for the genotype groups. In both clusters belonging to genotype IIa there was an identical substitution in urease alpha subunit-coding loci (G270C), but the two strains of MST cluster 11 also showed a mutation in *prpA*. Again, the two isolates of MST cluster 9 were unique, displaying mutations in *ure* and *prpA* which are not shared by other strains. The genotype IIf isolate Bm-GRC-B9h also exhibited unique mutations in *ure* and *vceC*. Except for the latter strain, all isolates for which mutations in these loci were found, have been isolated from sheep.

**Table 4:** Base substitutions and resulting amino acid changes in six predicted virulence-associated loci of Greek *B. melitensis* strains, relative to the reference strain *B. melitensis* 16 M. Loci designation, base numbers, and amino acid numbers refer to the reference genome. Cluster denomination according to cgMLST results

		*ure*			*bspB*	*prpA*	*vceC*
**Cluster/ strain**		BME_ RS03220	BME_ RS03225	BME_ RS03230	BME_ RS06340	BME_ RS01255	BME_ RS04715
MST11	Subst.	G270C	-	-	-	C222A	-
	Effect	silent				stop codon	
MST7	Subst.	G270C	-	-	-	-	-
	Effect	silent					
MST4	Subst.	-	-	-	C281T	-	-
	Effect				Gly94Asp		
MST10	Subst.	C1110T	-	-	-	-	-
	Effect	Met370Ile					
MST9	Subst.	-	-	C25T	-	del: 452–582	-
	Effect			Asp9Asn		truncated	
Bm-GRC-B9h	Subst.	-	G228A	-	-	-	G417C
	Effect		silent				Lys139Asn

### Genotyping of ***B. abortus*** Ba-GRC-O1c

**Fig. 5:**
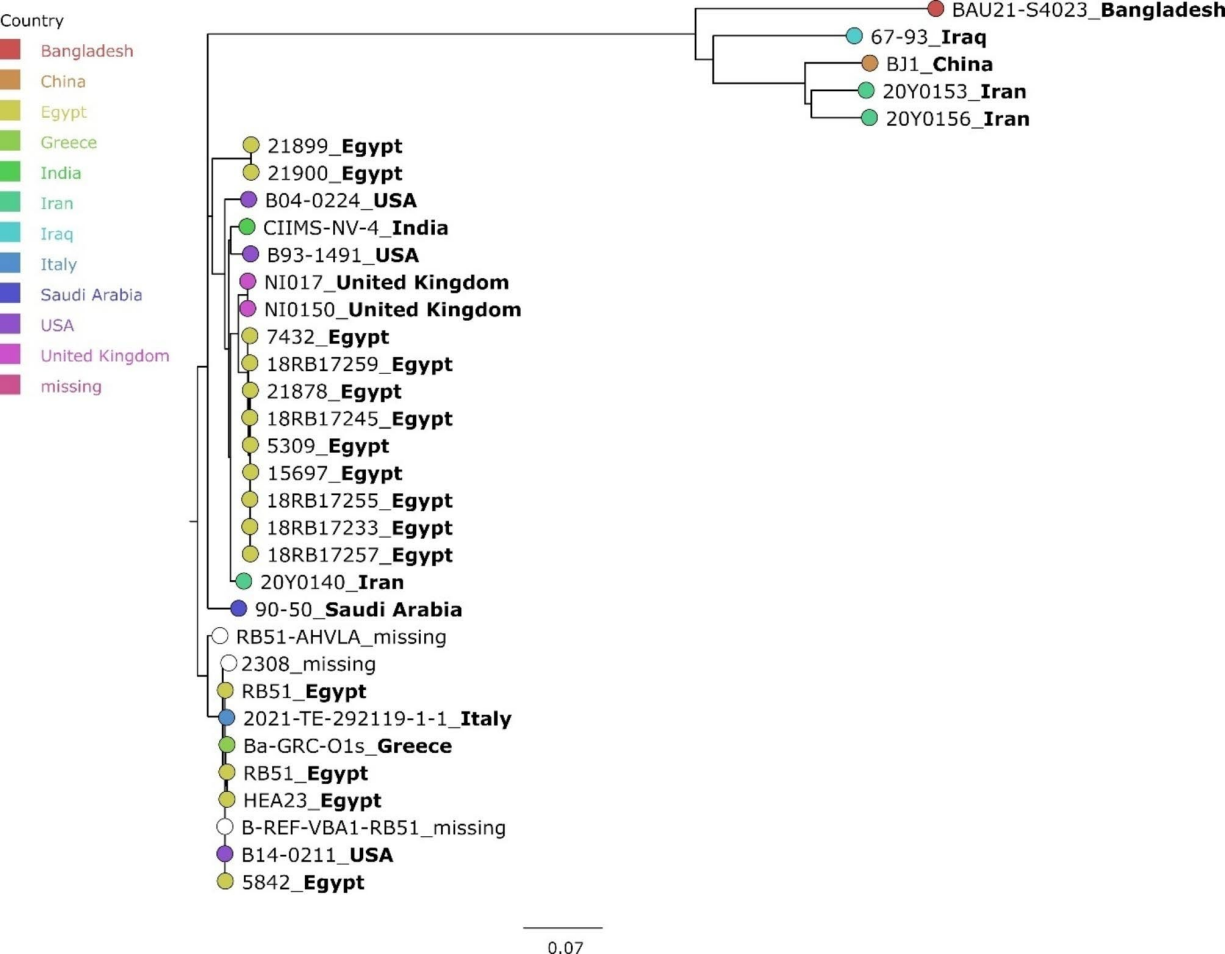
Maximum likelihood tree based on cgSNP alignment of *B. abortus* from Greece and foreign strains. The scale bar gives the number of base substitutions per site. For each strain, the country of origin is given by coloured branch tips and appended to the strain name

As shown by MLVA, the Greek *B. abortus* isolate Ba-GRC-O1c exhibited a higher similarity to *B. abortus* 2308 than to other Greek isolates. Thus, a SNP analysis was conducted including this *B. abortus* strain, its non-virulent descendant *B. abortus* RB51 and other field strains isolated mainly in Egypt (Fig. 5). Again, the Greek isolate clustered with the 2308 and RB51 strains, exhibiting 24 SNPs difference to the reference strain 2308 and merely 5 to 6 SNPs difference to the RB51 strains, except for RB51-AHLVA, to which 72 deviating SNPs were identified. To prove the identity of Ba-GRC-O1c as the RB51 vaccine strain, an *in silico* PCR for the BMEI0998 locus of the Bruce ladder PCR was conducted. However, it was found that the assembly broke at this specific locus, i.e. no contiguous sequence could be generated at this point by the assembler. Thus, the reads and the contigs were aligned to the assembly of *B. abortus* RB51 to check the read coverage at this locus. There was no drop in coverage at this position, however, the majority of reads had a low mapping quality, meaning that they mapped equally well to another position in the genome, hinting at a sequence duplication.

## Discussion

Although brucellosis is a notifiable disease in most European countries and most EU Member States are considered brucellosis free [4], the disease is still endemic, especially in the Mediterranean region [1, 47]. Greece, in particular, repeatedly reports high incidences of brucellosis in humans with the main causative agent being *B. melitensis* [6, 7, 19] and domestic ruminants as the main natural reservoir [19]. The results of the present study substantiate these findings, although, with one exception, no epidemiological connection between isolates from humans and small ruminants could be drawn from the collected isolates. This lack of connection was largely to be expected, as the human isolates came from different parts of the country than the animal isolates, i.e. the present results do not demonstrate a frequent transmission and mixing of *Brucella* populations between northern and southern Greece. Further, it can be expected that numerous brucellosis cases have not been detected and corresponding outbreak strains have been missed, due to lack of expertise, funding, facilities or personnel, and the reliance solely on seroepidemiological investigations which can be performed at a lower cost. A thorough epidemiological investigation of each human case by the time of diagnosis would be required to unambiguously establish the source of the infection. Two isolates from goats both are highly similar to isolates from sheep, so that in these cases an identical infection source or interspecies transmission can be assumed.

In most European countries, human brucellosis cases are either associated with travelling to endemic regions or consumption of contaminated dairy products [5, 11]. For Greece, it is known that human infections are mostly domestically acquired and even a connection to religious festivities has been suspected [4, 6]. Despite missing epidemiological information for the here investigated cases, the results of the study prove a strong association between several of the reported human infections, likely representing the continuous circulation of *Brucella* strains in an endemic area. It should be noted that the part of Greece from which the human isolates originate is an area of intensive sheep and goat farming. Thus, the probability of transmission due to profession increases, especially when using traditional herding techniques. There is no direct evidence that the number of human isolates correlated with the number of people positively diagnosed at the time of isolation based on the data of this research. Thus, it would be difficult, although not impossible, to correlate these cases with commercially sold dairy products, as it has been seen recently with camel milk in Israel [12], but it seems more probable that these cases could be the result of human-animal interaction. Yet, one human isolate clustering with Portuguese strains could represent an imported brucellosis case, perhaps by travelling of the patient, as it was the only representative of the IIf subgenotype which is often found in China, possibly originating from Pakistan [20].

The *B. melitensis* polytomy is divided into four main genotype lineages. In agreement with other studies, the West Mediterranean linage was basal to the other lineages in the phylogenetic tree [14, 48]. All of the here investigated *B. melitensis* isolates belonged to the East Mediterranean lineage, which is most common in Turkey [49]. So far, this lineage comprised nine subgenotypes with type IIa as the basal group. However, two of the Greek strains constituted a new, yet undiscovered subgenotype, which was basal to IIa, adding a tenth subgenotype to this polytomy. Pisarenko et al. [48] hypothesized that the divergence of IIa from other genotypes occurred in the second half of the 8th Century. Thus, it can be expected that this new subgenotype diverged before that time. As no similar foreign strains were found, the geographical extension and relevance of this subgenotype remain elusive.

The results further demonstrate the genetic diversity of the Greek *B. melitensis* community. The high similarity of strains within the clusters, e.g. low number of allelic differences in cgMLST analysis, despite an up to 10 years interval between sampling points, could account for the fact that a strain circulated undetected over a long period of time and that there was a persistent source of infection. The MLVA-based comparison additionally proved a high similarity to older Greek strains, e.g. isolated from cattle in 1990 [50], substantiating the persistence of this lineage in Greece and that these infections were caused by an endemic strain and were not imported cases, as it is seen in other European countries [5, 11]. In the analysed dataset, this IIb subgenotype was the most abundant. However, as comprehensive metadata was missing, it is indeterminable whether this genotype is representative for all of Greece or merely a certain region. Furthermore, it is well known that MLVA results can generally be affected by homoplasy and that WGS-based methods allow a more stable analysis of phylogenetic relationships [51, 52], so the results should be interpreted with caution.

Within the IIb genotype, clusters comprising Greek strains and strains from the Western Balkans were most prominent, proving a close phylogenetic relationship between *B. melitensis* strains circulating in South East Europe. According to Pappas [2], brucellosis was exclusively recognized in this region in Greece and parts of Turkey in 1990, while by 2010 brucellosis could be found in the majority of Balkan countries. Whether political changes during this 20-year period facilitated the spread of the disease from Greece to neighbouring countries or whether the existence of brucellosis was denied before that time is not known [2]. Further, it was reported that brucellosis was reintroduced to Bulgaria from Greece by two Bulgarian workers that worked as animal caretakers in Greece [53]. At present, the here investigated isolates represented the highest local genetic diversity of *B. melitensis* in this region. Brucellosis is a ubiquitous disease among bovines and small ruminants in Albania [54, 55]. Illegal trafficking of animals across borders, e.g. between Albania and Greece, has been recognized as one risk factor for the spread of brucellosis [1, 8]. However, in the SNP-based tree, there was no mingling of isolates from Greece with strains from the Western Balkans, that would account for a constant exchange of *B. melitensis* strains across the borders. If that was the case, the isolates would rather be expected to form a single cluster. For a more thorough investigation of the prevalence of certain genotypes, a comprehensive study of the *B. melitensis* communities from South East European countries is needed, as there is a lack of publicly accessible genomic data. For example, although brucellosis is endemic in North Macedonia [1], no publicly available WGS data have been deposited yet.

Being a zoonotic pathogen and biothreat agent of the category B [56], antimicrobial resistance and virulence genes of *Brucella* spp. are of particular concern [57]. As no AMR genes were detected in the Greek isolates, we focused on the housekeeping gene *rpoB*, where mutations can potentially confer resistance to rifampin [22, 23]. Further, *rpoB* had already been identified as highly specific and sensitive marker, which cannot only be used for genotyping of *Brucella* but also for species differentiation and assessment of the taxonomic composition of bacterial communities [13, 58–61]. The detected nucleotide substitutions in *rpoB* of the Greek isolates were in accordance with the clustering of the strains in the major genotype lineages, as has been seen before [13]. Further, the novel subgenotype of the East Mediterranean lineage, represented by MST cluster 9, could also be distinguished from the other strains of this genotype by exclusively harbouring the mutual SNP at gene position 3279 but no further mutation, in contrast to the other strains that at least exhibited one additional nucleotide change. The other mutations were in accordance with the findings of Georgi et al. [13] who identified specific *rpoB* nucleotide substitutions for the subgenotypes of the East Mediterranean lineage. Although rifampin resistances has not been screened in vitro for the here investigated strains, it can be expected that these mutations in *rpoB* did not affect the resistance, as identical mutations did not change the resistance of other *Brucella* strains before [13].

As in the case of *rpoB*, characteristic mutations for genotype lineages have been detected in some virulence-associated genes before [62]. In the Greek *B. melitensis* strains, the *ure* gene cluster displayed the highest number of mutations, which were in accordance with nucleotide substitutions identified in strains of genotype II [62]. Since no additional mutations were detected in isolates of human origin, the detected mutations might not increase the zoonotic potential of *B. melitensis*, as more human cases would be expected with strains harbouring a particular mutation. For the two strains of the novel subgenotype, two unique mutations could be identified which could help to distinguish this subgenotype from the others in the future, even without a WGS-based approach, e.g. by PCR.

As Greece is not officially free from bovine brucellosis [4] and there are reports of porcine brucellosis in swine herds from Greece as well as among the wild boar and hare population in the Balkan region [63–65], it can be assumed that *B. abortus* and *B. suis* are also circulating in Greece. However, the distribution and population structure have not yet been studied. Out of the 44 investigated isolates, one isolate was identified as *B. abortus* and the SNP typing proved this strain to be the *B. abortus* RB51 vaccine strain, which is a rifampin-resistant derivative of *B. abortus* 2308 [66]. From the data examined here, no conclusions can be drawn as to whether the strain caused the abortion in the animal. The failure of the *in silico* PCR for locus BMEI0998 (*wboA*) due to the break in the assembly, could be caused by an IS711 element disrupting *wboA* in RB51, which differentiates this strain from its ancestor [67], but also poses a challenge for genome assemblers as the *B. abortus* genome harbours multiple *IS711* copies [67, 68]. *B. abortus* RB51 has been used as a vaccination strain in cattle for many years, but it still has zoonotic potential and human brucellosis cases due to RB51 exposure have been observed [69, 70]. In Greece, the *B. melitensis* Rev-1 strain is used for the vaccination of small ruminants [7]. However, cases of illegal use of RB51 are known from other countries, like Italy, where RB51 was detected in the milk of a water buffalo [71]. This poses a risk to human health, especially if dairy products made from raw milk are sold and circulated [69].

## Conclusions

The potential misuse of a vaccine strain highlights problems of brucellosis control in Greece that probably promotes the prevalence of the disease in this region: an inadequate implementation of control and eradication programs, but also lack of health education [1, 8]. The fear of economic losses in the case of animal slaughtering further promotes the persistence of brucellosis foci [1]. Due to underreporting of brucellosis in Greece [8, 9], it can be expected that the real economic impact is greater than currently estimated, e.g. the costs of hospitalizations of patients covered by the statutory health insurance funds [8]. Although further research with more isolates covering the totality of the mainland and islands would give a much better picture, the present study has shown that several *B. melitensis* lineages circulate in Greece. These are mostly of endemic origin and persist in different foci. The endemicity of these strains suggests that stricter application and control of preventive measures are needed for controlling brucellosis in Greece.

### Electronic supplementary material

Below is the link to the electronic supplementary material.

**Additional file 1: Table: Sequencing quality data**. Quality metrics of sequencing data and de novo assemblies of the investigated strains, including affiliation to MLST sequence types and cgMLST clusters.

**Additional file 2: Table: MLVA profiles**. MLVA profiles of the investigated Greek strains, determined in silico, and entries from MLVAbank for Brucella strains isolated in Greece.

**Additional file 3: Figure: MLVA minimum spanning tree**. Minimum spanning tree based in the MLVA profiles given in Additional file 2. The newly sequenced Greek strains are coloured in red. For better visibility, the names of some clusters are given not directly in the circles but in the vicinity and dotted lines indicate the corresponding cluster. Numbers on the branches give the number of differing alleles

**Additional file 4: Table: Foreign strains used in this study**. Metadata and accession numbers for foreign strains used in this study

## Data Availability

The datasets generated during and analysed during the current study are available in the European Nucleotide Archive (ENA) repository, under the project number PRJEB56772.

## List of abbreviations

cgSNP: Core genome single nucleotide polymorphism
cgMLST: Core genome
MLVA: Multilocus variable number of tandem repeats analyses
MLST: Multilocus sequence typing
PCR: Polymerase chain reaction
SNP: Single nucleotide polymorphism
ST: Sequence type
WGS: Whole-genome sequencing
