# Supplementary figures and images for: Genotype diversity of brucellosis agents isolated from humans and animals in Greece based on whole-genome sequencing
Source: BMC Infect Dis. 2023 Aug 14;23:529. doi: 10.1186/s12879-023-08518-z (PMC10426126; doi:10.1186/s12879-023-08518-z)

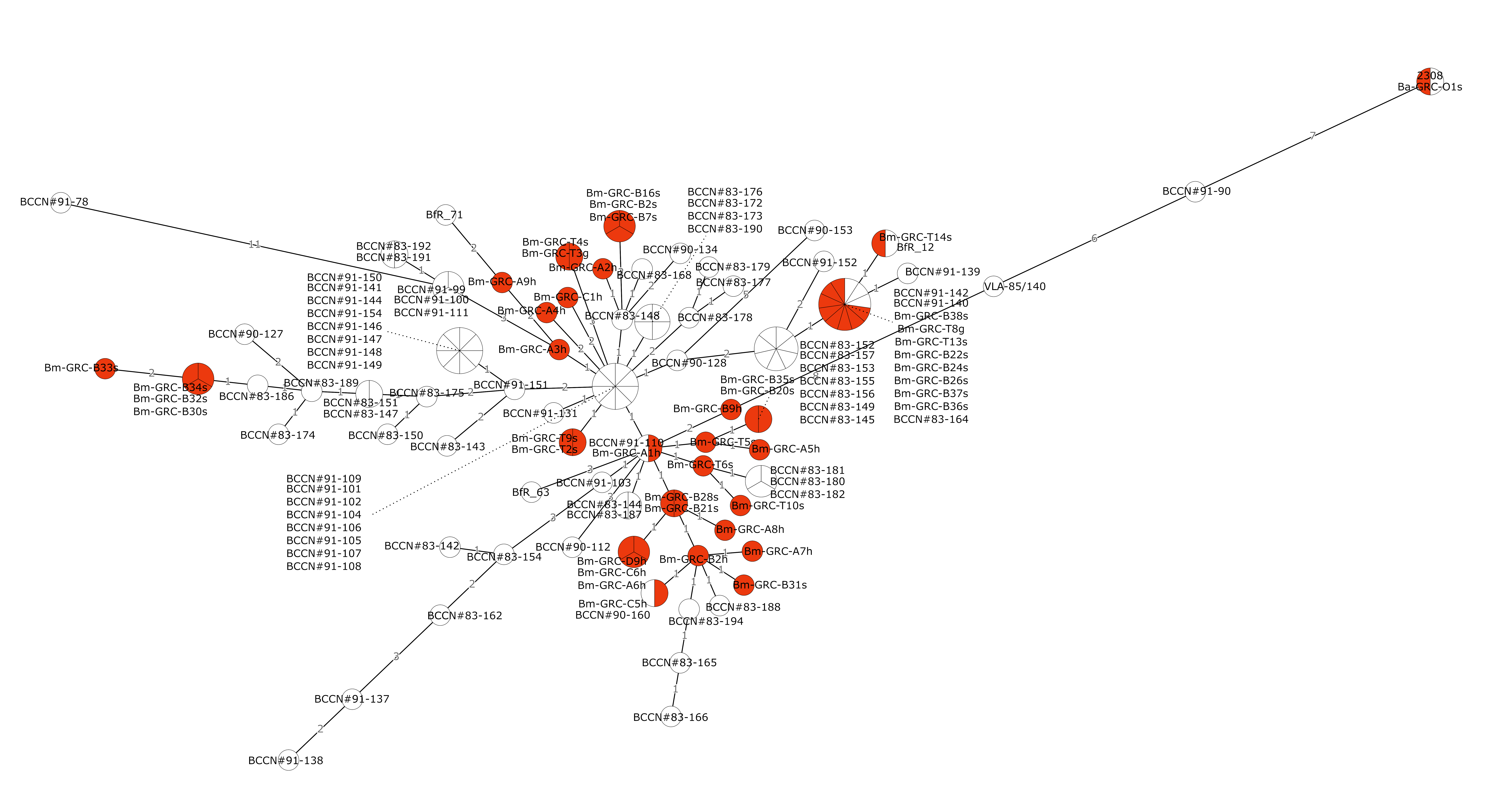

Supplement: Supplementary file 3 — Additional file 3: Figure: MLVA minimum spanning tree. Minimum spanning tree based in the MLVA profiles given in Additional file 2. The newly sequenced Greek strains are coloured in red. For better visibility, the names of some clusters are given not directly in the circles but in the vicinity and dotted lines indicate the corresponding cluster. Numbers on the branches give the number of differing alleles [file 12879_2023_8518_MOESM3_ESM.jpeg]
